# Genetic variability and diversity analysis for some agronomic traits of a sweet potato (*Ipomoea batatas* L.) collection: Insights for breeding superior genotypes

**DOI:** 10.1016/j.heliyon.2024.e38616

**Published:** 2024-09-27

**Authors:** Zakaria Alam, Md Anwar Hossain Khan, Md Iqbal Hossain, Md Rezaul Karim, Hasib-Bin Saif, A.A.M. Mohammad Mustakim, Md Mosharraf Hossain Molla, Md Monirul Islam, Sohela Akhter, Sanjida Akter

**Affiliations:** a Tuber Crops Research Centre, Bangladesh Agricultural Research Institute (BARI), Gazipur, 1701, Bangladesh; b Horticulture Research Centre, Bangladesh Agricultural Research Institute (BARI), Gazipur, 1701, Bangladesh; c Planning and evaluation wing, Bangladesh Agricultural Research Institute (BARI), Gazipur, 1701, Bangladesh; d Plant Physiology Division, Bangladesh Agricultural Research Institute (BARI), Gazipur, 1701, Bangladesh; e Entomology Division, Bangladesh Rice Research Institute (BRRI), Gazipur, 1701, Bangladesh

**Keywords:** Sweet potato, Genetic diversity, PCA, Cluster analysis, REML, Heritability, Selection accuracy, Genetic gain, Trait uniqueness, MGIDI

## Abstract

A study was conducted during the 2017 cropping season to assess the genetic variability of selected agronomic traits in sweet potato. A total of 355 sweet potato genotypes (351 from different countries of the world and four check varieties) were evaluated at Tuber Crops Research Sub Centre, Bangladesh Agricultural Research Institution, Bogura, Bangladesh. The experiment was conducted using an augmented randomized complete block design. Findings revealed positive correlations among five agronomic traits, namely the weight of marketable storage root per plant (MRW), weight of storage root per plant (RW), fresh weight of foliage per plant (FW), number of marketable storage root per plant (MRN) and number of storage root per plant (RN) suggesting their suitability for phenotypic selection. There was a positive skewness in most studied traits (FW, MRW, MRW and RW), indicating the presence of a few genotypes with exceptionally high values for those traits. By employing principal component analysis, the most influential traits in sweet potato genotypes were identified for first two components (Dim) showing significant contributions to Dim-1 and Dim-2 collectively accounting for 84.7 % of the total variance. Through cluster analysis, two main clusters (Cluster I and Cluster II) were identified among the studied genotypes, each characterized by distinct trait patterns. Cluster I demonstrated superior performance in all traits, while Cluster II exhibited lower values for all traits. Further sub-clustering within Cluster II (sub-clusters IIA and IIB) revealed additional variations of the collection. The Euclidean distance for inter-cluster (ranging from 7.94 to 11.39) and intra-cluster (ranging from 3.17 to 10.38) were determined utilizing the complete distance method. In restricted maximum likelihood (REML) model, all the traits were significantly influenced by genetic effects, with broad-sense heritability ranging from 32 % to 70 %. The REML displayed high selection accuracy, with values exceeding 91 % for studied traits. The Multi-Trait Genotype-Ideotype Distance Index (MGIDI) identified 18 genotypes, with Moz1.15 emerging as the best-performing genotype under a 5 % selection pressure. Among the selected genotypes, trait uniqueness was highest for FW (54 %), followed by RN (50 %), MRN (18 %), MRW (14 %), and RW (5 %). The selection gain for these top-ranked genotypes varied between 23.2 % and 69.2 %. Findings established the existence of useful genetic variability for selected agronomic traits of sweet potato that could be exploited for breeding, genetics study and conservation.

## 1 Introduction

Plant genetic resources, regarded as one of the most crucial natural assets, have been the central focus of extensive investigation, leading to notable advancements in the field [1]. The main goal of gene banks is to preserve the genetic diversity of crop resources, with growing attention being given to the conservation of plant genetic resources today [2]. Understanding genetic diversity both within and among populations is essential for developing effective and efficient conservation strategies for plant genetic resources [3].

Sweet potato [*Ipomoea batatas* (L.) Lam] is a dicotyledonous plant belonging to the Convolvulaceae family, also known as the morning glory family. It ranks as the third most important root and tuber crop globally, following potato (*Solanum tuberosum* L.) and cassava (*Manihot esculenta* Crantz) [4]. It originates from northwestern South America and may have emerged from either a hybrid cross or karyotypic changes of an unidentified plant belonging to the genus *Ipomoea*. The domestication of this root vegetable is closely linked to the emergence of agricultural communities in the Tropical Forest region around 2500 B.C. Subsequently, the Spanish introduced it to Europe, where it subsequently spread to China, Japan, Malaysia, and the Moluccas region. Furthermore, the Portuguese were responsible for its introduction to India, Indonesia, and Africa [5]. Over time, there has been a global increase in the cultivation of this specific crop, as evidenced by the latest data from the International Potato Centre (CIP), which reveals an annual worldwide production of 150 million tons of sweet potato root. The majority of this production, which amounts to more than 77.38 million tons, is contributed by China, along with other Asian countries such as Indonesia, Japan, and Korea [6].

The introduction of sweet potato into Bangladesh occurred in the latter part of the nineteenth century [7]. Historically, the consumption of sweet potatoes in Bangladesh has been closely linked to economically disadvantaged communities. Due to its affordability and ease of cultivation, this "crop of the poor" has become a dietary staple in impoverished households. Currently, the exceptional qualities of sweet potatoes, including their high production levels, palatability, and crude protein content, further enhance their significance [8]. The production of sweet potatoes in Bangladesh displayed a positive trajectory over the previous decade, with a notable surge of 16 % in the overall yield between 2018-19 and 2020–21 [9]. The Tuber Crops Research Centre (TCRC) of Bangladesh Agricultural Research Institute (BARI) is actively involved in addressing this overall yield advancement matter by introducing high-yielding varieties, as well as the promotion of their technologies and the enhancement of farmer awareness. BARI has successfully released seventeen high-yielding varieties of sweet potato. These varieties have been developed through the half-diallel cross breeding method and the selection from both domestic and international germplasm collections [10], with a primary focus on the importance of genetic diversity and variability.

Sweet potato is a highly heterozygous, polyploid plant that is typically self-incompatible and propagated vegetatively, presenting several challenges for conventional breeding techniques. Research aimed at improving sweet potato has often been overshadowed by the emphasis on major cereal crops and other export cash crops. However, speeding up the breeding of sweet potato is essential to address the demands of an increasing global population and to support sustainable agriculture [11]. This acceleration depends heavily on studies of genetic and trait diversity. The remarkable traits that govern the ability to adapt to diverse ecological and geographical conditions ultimately display a broad range of variations in terms of their agricultural traits and the morphology of their roots and shoots [[12], [13], [14], [15], [16]]. This variation underscores the significant potential for crop enhancement, as assessing crop diversity offers a solid basis for choosing parental lines and developing a breeding program, thereby delivering both a framework and a strategic approach. Moreover, advances in linking genetics with morphological variations have greatly enhanced the breeding of major crops [17,18], and sweet potato can similarly take advantage of this information. This involves identifying genes that regulate important agronomic traits and utilizing advanced breeding techniques. However, there remains a scarcity of genetic resources for sweet potato breeding, which limits the application of modern crop improvement tools like high-throughput phenotyping, genome editing and genomic selection.

Selecting the top-performing genotypes from a large gene pool is challenging, but principal component analysis (PCA) is a method employed to reduce the magnitude of multivariate datasets from extensive collections [12,[19], [20], [21]]. On the other hand, mixed models are frequently employed to enhance selection efficiency [16,[22], [23], [24], [25], [26], [27], [28]]. However, understanding the genetic mechanism governing trait inheritance and the influence of genetic factors on their expression is indispensable for the success of a breeding program [17,29]. An effective approach for analyzing and estimating genetic parameters using agronomic traits is through restricted maximum likelihood (REML) model [30]. It has been employed in diverse crop breeding initiatives, including sweet potato [31], white Guinea yam [32], soybean [33], etc. To identify the top-performing genotypes, a new method known as the multi-trait genotype ideotype distance index (MGIDI) is being utilized across various crops, including sweet potato [12,16,25,28,32,34].

The objective of this study was to evaluate genetic diversity of 351 collected sweet potato genotypes and identify superior ones using multivariate analysis. Additionally, the research analyzed the variance components of these genotypes to inform future sweet potato breeding programs. The findings will contribute to genetic research efforts aimed at improving sweet potatoes through agronomic traits.

## 2 Materials and methods

### 2.1 Description of experimental sites

The research was conducted at the Tuber Crops Research Sub Centre of the Bangladesh Agricultural Research Institute in Bogura, Bangladesh. The site coordinates are 23.6850° N latitude and 90.3563° E longitude, with 10 m elevation in the coastal region and 105 m in the northern area. The soil in this area is sandy loam with a pH of 6.23. It contains 0.10 % nitrogen and 3.5 % organic matter. The available phosphorus concentration is 28 ppm, while the exchangeable amounts of calcium, potassium, magnesium, sodium, iron, manganese, copper, and zinc are measured at 0.16, 0.60, 0.10, 1.52, 28, 6.32, 0.23, and 0.74 meq per 100 g of soil, respectively [6].

### 2.2 Experimental materials, layout and design

A detailed description of 351 collected sweet potato genotypes, along with four check varieties, is presented in Table 1 and Table S1, along with a description of the climate conditions at the collection sites. The trial was organized into 10 blocks using an augmented randomized block design, where each block contained 35 unique genotypes without replication, except for one block that had 36 genotypes, along with four replicated control checks. For each genotype, 10 vines were planted in a single row.

**Table 1:** A comprehensive overview of 355 genotypes of sweet potato and detailed climate description of the source of collections.

Number of genotypes	Country of source	Altitude (m)	Average rainfall (mm)	Average temperature (⁰C)	Humidity (%)	Average sunshine (h)
126	Mozambique	345	109.73	25.62	71.59	10.59
57	Japan	438	84.47	15.19	76.09	9.92
28	Indonesia	367	136.87	27.11	79.69	10.56
140^+^^a^	Bangladesh	105	102	27.74	66.29	10.65

a check varieties: BARI Mistialu-12, BARI Mistialu-8, BARI Mistialu-15 and BARI Mistialu-14.

### 2.3 Crop husbandry, harvesting and collection of data

The cultivation techniques for sweet potato were carried out according to the procedure outlined by Alam et al. [6]. Five plants were sampled from each genotype during harvesting, which was conducted 130 days after vine planting. Data were collected on various parameters, including the fresh weight of foliage per plant (FW) in grams, the number of storage roots per plant (RN), the weight of storage roots per plant (RW) in grams, the number of marketable storage roots per plant (MRN), and the weight of marketable storage roots per plant (MRW) in grams. These measurements followed the methodology described by Alam et al. [6].

### 2.4 Statistical analysis

The 351 genotype adjusted means including four checks of augmented design was calculated using “augmentedRCBD” package of R studio [35]. The trait correlation, PCA and cluster analysis were performed using those means using “corrplot”, "MASS", "factoextra", "tidyverse", ggplot2", devtools, "vqv/ggbiplot", "FactoMineR", "corrplot", “factoextra”, “ggplot2”, “NbClust” and “clv” packages of R studio. The likelihood ratio test (LRT) was conducted with a significance threshold of *p* < 0.05. Variance components for REML were assessed, along with factor analysis and the calculation of the MGIDI index. These analyses were completed using the "metan" package in R Studio [35], applying a selection intensity of 5 % with a positive sense for all studied traits.

## 3 Results and discussion

### 3.1 Source wise performance of sweet potato collection

**Fig. 1:**
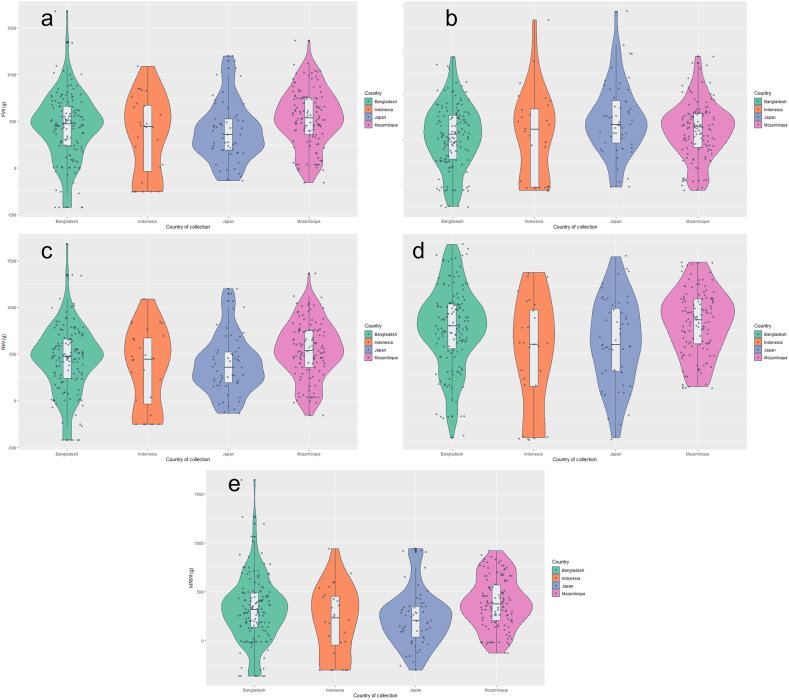
Country wise performance of sweet potato collections in fresh weight of foliage per plant (FW) (a), number of storage root per plant (RN) (b), weight of storage root per plant (RW) (c), number of marketable storage root per plant (MRN) (d) and weight of marketable storage root per plant (MRW) (e).

The violin plots represent the source wise genotype performance in FW, RN, RW, MRN, and MRW traits (Fig. 1). A violin plot is a graphic that combines a box plot and a density plot. In Fig. 1, the density plots are on both sides of the box plot, showing symmetrical distributions of different traits. The y-axis shows the range of values for sweet potato traits while the x-axis represents different source of collections. The outliers are values beyond defined range of whiskers. Median is far from upper edge of the box, indicating scattered data points above the median. One whisker longer than the other suggests skewed distribution. Majority of values clustered towards upper end is positively skewed. Mean is significantly affected by extreme values in positively skewed distribution. Conversely, the median is not strongly impacted by a few extreme values. Fig. 1 illustrates that most traits, with the exception of MRN (Fig. 1d), display positive skewness, indicating the presence of a small number of extremely large values for FW, RN, RW, and MRW. Concerning FW, the distribution of large values was most pronounced in Bangladeshi collections, followed by Mozambique, Japan, and Indonesia (Fig. 1a). Likewise, for RN (Fig. 1b), the Japanese collections exhibited the highest distribution value, followed by Indonesia, Bangladesh, and Mozambique. In terms of RW, Bangladesh demonstrated superior outcomes, followed by Japan, Mozambique, and Indonesian collections (Fig. 1c). MRW (Fig. 1e) showcased the highest level of high value distribution in Bangladeshi collections compared to the others. According to Javed et al. [36], the importance lies in the distribution within a particular genotype group when engaging in the process of selecting from an extensive assemblage. This distribution facilitates the identification and assimilation of advantageous genetic variations.

### 3.2 Correlation among agronomic traits

**Fig. 2:**
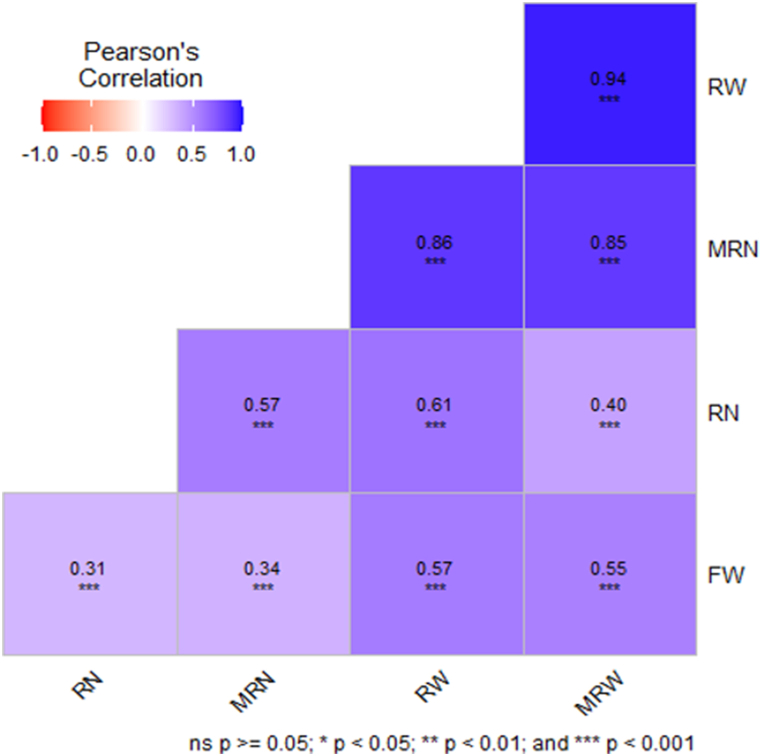
Co-efficient of correlation between weight of marketable storage root per plant (MRW), weight of storage root per plant (RW), fresh weight of foliage per plant (FW), number of marketable storage root per plant (MRN) and number of storage root per plant (RN) of 355 sweet potato genotypes.

Fig. 2 displays the correlation coefficients among the five agronomic traits examined. The results showed considerable positive associations (*p* < 0.05) relating to all the examined characteristics. The coefficients ranged from 0.31 to 0.94. In several studies conducted by Alam et al. [6,12,16,25,26] on various sweet potato genotypes, similar types of correlations were discovered among yield traits, linking these traits to the identification of the most high-performing sweet potato genotypes. Given the lack of substantial negative correlations identified in the traits examined in the current study, it can be inferred that these traits are more predisposed to being transmitted to the subsequent generation. Consequently, this facilitates enhancement through phenotypic selection in the event of the existence of high heritability [37].

### 3.3 Principal component analysis (PCA)

**Fig. 3:**
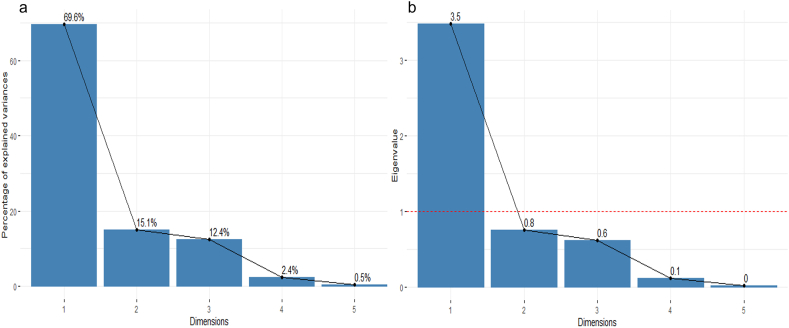
Proportional variation (a) explained by five axes and their eigenvalues (b).

In the present investigation, a multitude of features have been identified to differentiate genotypes that are useful for genetic diversity analyses of sweet potato through principal component analysis (PCA). There was a total of five principal component axes identified at the beginning, specifically Dim-1, Dim-2, Dim-3, Dim-4, and Dim-5 (Fig. 3). Among these, Dim-1 had eigenvalues that reached a maximum of 1.0 (Fig. 3b). This particular axis accounted for a variance of 69.6 % (Fig. 3a.). The first two principal components, referred to as Dim-1 and Dim-2, collectively accounted for 84.7 % of the overall variance and had eigenvalues of 3.5 and 0.8, respectively (Fig. 3a).

This implies that the traits identified in these two initial axes exhibit a strong correlation and variation among the genotypes. The utilization of the initial two axes for the exploration of utmost variation and the utilization of PCA in conjunction with those axes has been discovered to be exceedingly efficient in the detection of pivotal attributes for the efficient phenotypic portrayal of sweet potato, as stated by Palumbo et al. [38] and Alam et al. [12].

**Fig. 4:**
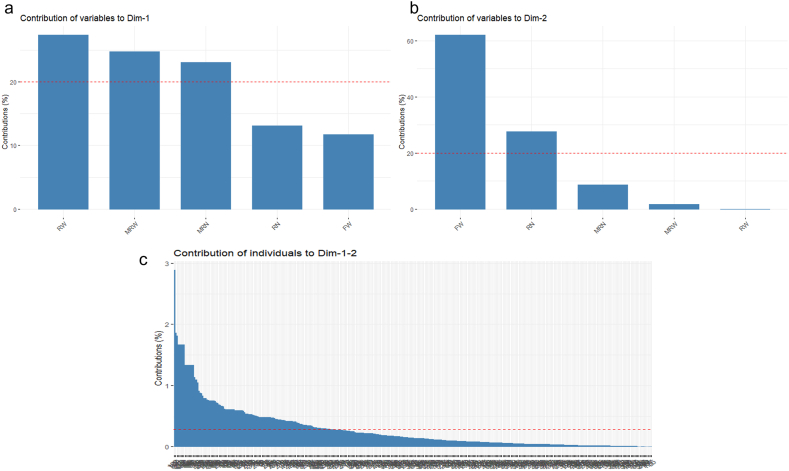
Contributions of studied traits in first principal component (a), second component (b) and contribution of genotypes (c) to first two principal components in PCA. ^MRN^ number of marketable storage root per plant, ^RN^ number of storage root per plant, ^MRW^ weight of marketable storage root per plant, ^RW^ weight of storage root per plant and ^FW^ fresh weight of foliage per plant.

In a dissection of significant principal components, RW exhibited the most significant contribution, exceeding 25 %, in the first principal axis (Fig. 4a). This was followed by MRW and MRN. Conversely, FW made a contribution of over 60 %, followed by RN, to the second principal axis (Fig. 4b). One third of the total studied genotypes were at the critical level of contributing to the first two principal axes (Fig. 4c). This implies that it may be possible to select genotypes from this subset of total genotypes, as they have the highest contribution to the first two principal axes. Traits that exhibit substantial correlations with a principal component will exert a more pronounced influence on that particular component, whereas traits with feeble correlations will exert a diminished influence. This phenomenon facilitates the identification of pivotal traits that account for the highest degree of variability in the data and may be harnessed for subsequent analytical or selective endeavors [[39], [40], [41]].

**Fig. 5:**
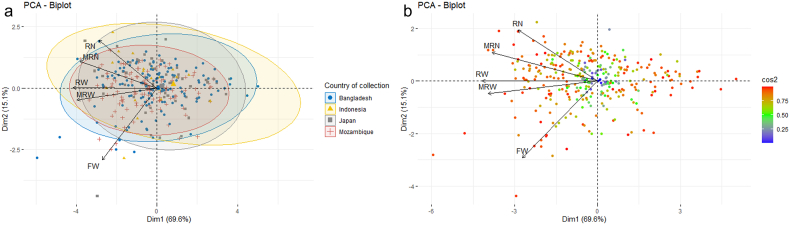
Genotype grouping based on source of collection (a), correlation of studied traits and genotypes' contribution to first two principal components (b). ^MRN^ number of marketable storage root per plant, ^RN^ number of storage root per plant, ^MRW^ weight of marketable storage root per plant, ^RW^ weight of storage root per plant and ^FW^ fresh weight of foliage per plant.

The PCA biplot analysis entailed a detailed comparison of the eigenvalues of both Dim.1 and Dim.2, which were generated using principal component analysis (PCA), for traits, genotypes and source of collection as depicted in Fig. 5. Different source of collections was grouped in the biplot showing their diversity between groups and adherence within groups (Fig. 5a). The outcomes from the first two principal component axes, specifically Dim.1 and Dim.2, yielded a detailed overview of the complexity of variation between the plotted components, accounting for a total of 84.7 % of overall variability (Fig. 5). The vectors of traits (variables) displayed acute angles indicating positive correlation, while obtuse or straight angles indicated negative correlation, and right angles indicated no correlation (Fig. 5b). The proximity between genotypes is directly proportional to their similarity, and conversely, genotypes that are more similar have a shorter distance between them (Fig. 5b). A high positive correlation was discovered between the studied traits (Fig. 5b). Additionally, MRW, RW and MRN demonstrated their contribution vector to Dim.1 and RN and FW vector were placed to Dim.2 axis (Fig. 5b). The color gradient in Fig. 5b illustrates the extent to which genotypes contribute to the two principal components. The highest contributing genotypes are carrying red color (scattered outside of biplot) and lowest with blue (center of biplot) (Fig. 5b). Kaysar et al. [39] also conducted Principal Component Analysis (PCA) in order to identify superior genotypes and analyze the morphological traits. Moreover, several authors have confirmed that the traits’ correlation based on angle of eigen vectors in sweet potatoes provide valuable information and effectiveness for genetic manipulation and crop improvement programs in sweet potatoes [12,[42], [43], [44]], which is consistent with the present study.

In evident, by giving emphasis on these traits, breeders are able to make selections of superior clones for breeding purposes, ultimately enhancing the overall performance of sweet potato genotypes [45]. Patra [46] stated that the PCA is a significant tool for analyzing and comprehending plant traits and their associations with plant performance and selection criteria.

### 3.4 Cluster analysis

**Fig. 6:**
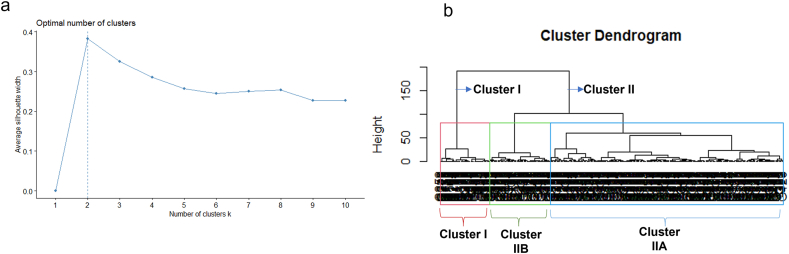
Optimum cluster identification (a) and cluster dendrogram for 355 collected sweet potato including checks.

According to the silhouette method, two optimal clusters, namely cluster I and cluster II, were identified based on the average performance of 355 genotypes across the studied traits (Fig. 6a). Additionally, two sub-clusters, labeled IIA and IIB, were identified within cluster II (Fig. 6b). The inter-cluster distance and intra-cluster distance can be found in Table 2. The value of the intra-cluster distance for cluster I, IIA, and IIB were found to be D^2^ = 4.84, D^2^ = 10.38, and D^2^ = 3.17, respectively (Table 2). The inter-cluster distance between cluster I and IIA was determined to be D^2^ = 7.94, between cluster I and IIB was calculated as D^2^ = 9.06, and between IIA and IIB was found to be D^2^ = 11.39 (Table 2). Cluster analysis identifies genotypes by grouping them based on their similarity in terms of traits which helps in determining the extent of genetic diversity among the genotypes through Euclidean distance [47]. For instance, Mohammed et al. [48] examined the diversity of 116 sweet potato genotypes by analyzing 17 agro-morphology and physicochemical traits. Their study identified 12 clusters, with Euclidean distances between clusters ranging from 2.04 to 11.00, which is in line with the present study. This indicates a significant genetic divergence between the clusters and demonstrates considerable heterogeneity among the accessions between clusters. The present study aligns with the findings of Bashyal et al. [49], who observed a similar intra-cluster distance. In addition, clustering of sweet potato genotypes based on morphological traits will uncover the presence of replicated entries and the grouping together of entries based on their genetic categories which will also help them to carry out an effective germplasm conservation program [50]. For instance, the hierarchical classifications are able to classify the genotypes into genetic groups and detect the existence of duplicate specimens [51].

**Table 2:** Inter cluster and intra cluster Euclidean distance (D^2^) between genotypes based on complete distance method.

	Cluster I	Cluster IIA	Cluster IIB
Cluster I	4.84	–	–
Cluster IIA	7.94	10.38	–
Cluster IIB	9.06	11.39	3.17

Cluster I comprise of 63 genotypes, while IIA and IIB consist of 241 and 51 genotypes, respectively (Table 3). The mean value of FW was determined to be the greatest in cluster I, with a measurement of 467.49g, and the smallest in cluster IIB, which measured 26g (Table 3). In the case of RN, the highest mean was observed in cluster I, with a value of 8.24, while the lowest was found in cluster IIB, measuring 0.10 (Table 3). Regarding RW, cluster I exhibited the highest mean at 896.6g, whereas the lowest value was observed in cluster IIB at −71.45g. The highest MRN was discovered in cluster I at 4.20, while the lowest was found in cluster IIB at −0.69. Lastly, the highest MRW was observed in cluster I, measuring 726.54g, and the lowest was in cluster IIB at −88.08g. The comparison of mean agronomic traits among clusters has been conducted by multiple researchers [12,[52], [53], [54]]. Their findings consistently indicate that evaluating the mean performances of identified clusters is an effective approach for selecting superior genotypes from a large collection.

**Table 3:** Genotype representation in clusters and mean performance of clusters.

Clusters	Number of genotypes	FW (g)	RN	RW (g)	MRN	MRW (g)
I	63	467.49	8.24	896.60	4.20	726.54
IIA	241	427.99	6.54	460.51	2.02	301.31
IIB	51	26.00	0.10	−71.45	−0.69	−88.08

^MRN^ number of marketable storage root per plant, ^RN^ number of storage root per plant, ^MRW^ weight of marketable storage root per plant, ^RW^ weight of storage root per plant and ^FW^ fresh weight of foliage per plant.

### 3.5 Analysis of genetic parameters and variance components using REML

The likelihood ratio test (LRT) revealed statistically significant differences (*p* < 0.05) across all traits—MRW, FW, RW, RN and MRN—among the genotypes studied. These findings provide supporting evidence that the genetic components and parameters obtained are not only consistent but also reliable. The REML-derived genotypic variance (σ^2^_g_) components indicate that genetic effects have a moderate to high influence on the traits FW, RN, RW, MRN, and MRW, with percentages of 69.9 %, 53.7 %, 39.1 %, 32.3 %, and 39.4 %, respectively (Table 4). Among the variance components, it was discovered that the genotypic variances (σ^2^_g_) surpassed the residual variances (σ^2^_e_) for the characteristics FW and RN, as demonstrated in Table 4. This implies the potential for advancements through the process of selection. The broad sense heritability (h^2^) of studied traits ranged from moderate to high, specifically falling between 32 % and 70 %. Broad sense heritability is a measure that calculates the fraction of phenotypic variance attributable to the variation in all genotypic effects, including additive, dominant, and epistatic influences [12,16,55]. Additionally, the heritability of genotype means (h^2^_mg_) exhibited a high value exceeding 82 %. Furthermore, the selection accuracy (r_gg_) for all the traits that were studied was discovered to be higher than 91 %. The estimation of heritability depends on the existence of sampling errors. A higher response to selection can be achieved if the accuracy values are increased; however, the estimation of accuracy in breeding values is still dependent on heritability, as confirmed by Visscher et al. [55]. Selection accuracy refers to the degree of correlation between the estimated genotypic values and their respective measurements. The coefficient of genotypic variation (CV_g_) showed a higher value for the FW trait compared to RN, RW, MRN, and MRW. An increased coefficient of genotypic variation signifies greater genetic variability among the genotypes under investigation. Conversely, the reduced values of the genotypic coefficient for certain traits indicate a significant environmental impact on their expression. Yield-related traits of sweet potato are affected by weather and soil conditions. However, it is important to recognize that the genotypic variance coefficient alone is not a reliable indicator of heritable variation and should be assessed alongside heritability estimates, as highlighted by Fotirić-Akšić et al. [29].

**Table 4:** Variance components of studied traits for 355 sweet potato genotypes.

Components	Traits				
	FW	RN	RW	MRN	MRW
LRT	20.5^a^	7.5^a^	21.7^a^	19.7^a^	24.7^a^
σ^2^_g_ (%)	69.9	53.7	39.1	32.3	39.4
σ^2^_e_ (%)	30.1	46.3	60.9	67.7	60.6
h^2^	0.70	0.54	0.39	0.32	0.39
h^2^_mg_	0.96	0.92	0.87	0.83	0.87
r_gg_	0.98	0.96	0.93	0.91	0.93
CV_g_	57.8	41.5	36.7	35.3	44
Overall Mean	392.30g	6.33	537.65g	2.55	398.17g

a Significant at 5 % probability level by chi-square (X^2^) test in likelihood ratio test (LRT), ^σ2g^ genotypic variance, ^σ2e^ residual variance,^h2^ broad sense heritability, ^rgg^ selection accuracy,^h2mg^ heritability of genotype average, ^CVg^ genotypic co-efficient of variation, ^MRN^ number of marketable storage root per plant, ^RN^ number of storage root per plant, ^MRW^ weight of marketable storage root per plant, ^RW^ weight of storage root per plant and ^FW^ fresh weight of foliage per plant.

### 3.6 Selection of superior genotypes

**Fig. 7:**
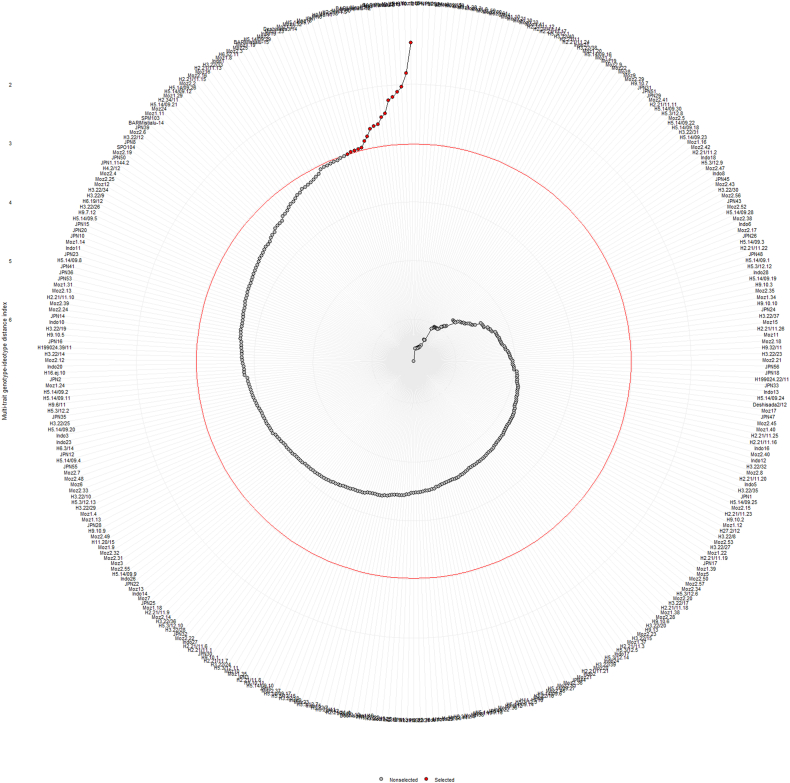
Ranking of superior sweet potato genotypes using MGIDI index.

In the factor analysis (Table 5), the predicted means for all evaluated traits in the selected sweet potato genotypes were greater than the observed means, leading to positive selection differentials for FW, RN, RW, MRN, and MRW of 283, 1.6, 270, 0.75, and 242, respectively. The selection gain percentage was also higher for all studied traits, ranging from 23.2 % to 69.2 %. The highest percentage of trait uniqueness was found in FW (54 %), followed by RN (50 %), MRN (18 %), MRW (14 %), and RW (5 %). Many research studies have shown that selection can leverage existing genetic variations to attain specific improvements and increase the value of particular traits, especially when the traits being selected are largely identical [12,16,56]. In Fig. 7, the red circular line represents the critical point for a 5 % selection intensity level based on the multi-trait genotype ideotype distance index (MGIDI). The red dots above this red circle indicate the selected genotypes. The MGIDI index identified the top-ranked genotype as Moz1.15, followed by H9.10.12, H5.ej.10, Moz2.11, Moz2.27, BARIMistialu-8, Moz1.7, JPN37, Moz1.26, BARIMistialu-12, Moz14, Moz2.30, H2.21/11.5, Moz2.58, H9.48.11, JPN6, JPN7, and Moz18 (Fig. 7). Several studies have successfully utilized the MGIDI index to select superior genotypes of sweet potato [12,16,25].

**Table 5:** Factor analysis of selected sweet potato genotypes for studied agronomic traits obtained using MGIDI index.

Traits	X_o_	X_s_	SD	SG (%)	TU (%)
FW	392	675	283	69.2	54
RN	6.33	7.92	1.6	23.2	50
RW	538	807	270	43.4	5
MRN	2.55	3.3	0.747	24.2	18
MRW	398	640	242	52.6	14

^Xo^ observed mean, ^Xs^ predicted mean, ^SD^ selection differential, ^SG^ selection gain, ^TU^ trait uniqueness, ^MRN^ number of marketable storage root per plant, ^RN^ number of storage root per plant, ^MRW^ weight of marketable storage root per plant, ^RW^ weight of storage root per plant and ^FW^ fresh weight of foliage per plant.

## 4 Conclusion

This study established the existence of useful genetic variability for selected agronomic traits of sweet potato that could be exploited for breeding, genetics study and conservation. Positive correlations were found among five key traits, and the observed positive skewness indicated that some genotypes had exceptionally high values. Principal component analysis identified the most influential traits, which together explained 84.7 % of the total variance, while cluster analysis revealed two main clusters with distinct trait patterns. All traits were significantly influenced by genetic effects, with broad-sense heritability ranging from 32 % to 70 %, and high selection accuracy was attained. The trait uniqueness of the selected genotypes highlights the extensive genetic resources of sweet potatoes for the studied traits. The best-performing genotypes were identified based on positive selection gains derived from the factor analysis of the MGIDI index. These results highlight the significant genetic diversity present in sweet potato, which can be utilized for breeding, genetics research, and conservation initiatives. The study was carried out in a single location, which might not account for environmental variability and genotype-by-environment interactions that could impact the performance of the sweet potato genotypes. This limitation may hinder the generalizability of the results to other regions with different growing conditions.

## Data availability statement

Data will be made available on request.

## CRediT authorship contribution statement

**Zakaria Alam:** Writing – review & editing, Writing – original draft, Validation, Supervision, Methodology, Investigation, Formal analysis, Data curation, Conceptualization. **Md Anwar Hossain Khan:** Supervision, Investigation, Conceptualization. **Md Iqbal Hossain:** Formal analysis, Data curation. **Md Rezaul Karim:** Formal analysis, Data curation. **Hasib-Bin Saif:** Formal analysis. **A.A.M. Mohammad Mustakim:** Formal analysis, Data curation. **Md Mosharraf Hossain Molla:** Supervision, Conceptualization. **Md Monirul Islam:** Data curation, Conceptualization. **Sohela Akhter:** Writing – review & editing, Supervision, Investigation, Conceptualization. **Sanjida Akter:** Formal analysis, Data curation.

## Declaration of competing interest

The authors declare that they have no known competing financial interests or personal relationships that could have appeared to influence the work reported in this paper.
