# Supplementary material for: Genetic variability and diversity analysis for some agronomic traits of a sweet potato (Ipomoea batatas L.) collection: Insights for breeding superior genotypes
Source: Heliyon. 2024 Sep 27;10(19):e38616. doi: 10.1016/j.heliyon.2024.e38616 (PMC11470407; doi:10.1016/j.heliyon.2024.e38616)
Supplement: Multimedia component 1 [file mmc1.docx]

Table S1. Collected 351 sweet potato genotypes including four checks with their country of collection

| Genotype | Source | Genotype | Source | Genotype | Source | Genotype | Source | Genotype | Source | Genotype | Source | Genotype | Source |
| --- | --- | --- | --- | --- | --- | --- | --- | --- | --- | --- | --- | --- | --- |
| BARIMistialu-8 | Bangladesh | Deshisada2/12 | Bangladesh | H16.ej.10 | Bangladesh | Indo7 | Indonesia | JPN28 | Japan | Moz22 | Mozambique | Moz2.48 | Mozambique |
| BARIMistialu-12 | Bangladesh | H2.34/11 | Bangladesh | H2.21/11.24 | Bangladesh | Indo8 | Indonesia | JPN29 | Japan | Moz23 | Mozambique | Moz2.49 | Mozambique |
| BARIMistialu-14 | Bangladesh | H9.13 | Bangladesh | H2.21/11.25 | Bangladesh | Indo9 | Indonesia | JPN30 | Japan | Moz24 | Mozambique | Moz2.50 | Mozambique |
| BARIMistialu-15 | Bangladesh | Deshisada3/14 | Bangladesh | H2.21/11.26 | Bangladesh | Indo10 | Indonesia | JPN31 | Japan | Moz25 | Mozambique | Moz2.51 | Mozambique |
| Deshisada1/19 | Bangladesh | H2.21/11 | Bangladesh | H9.10.1 | Bangladesh | Indo11 | Indonesia | JPN32 | Japan | Moz2.2 | Mozambique | Moz2.52 | Mozambique |
| H4.2/12 | Bangladesh | H11.15/10 | Bangladesh | H9.10.2 | Bangladesh | Indo12 | Indonesia | JPN33 | Japan | Moz2.3 | Mozambique | Moz2.53 | Mozambique |
| H3.22/31 | Bangladesh | H9.32/11 | Bangladesh | H9.10.3 | Bangladesh | Indo13 | Indonesia | JPN34 | Japan | Moz2.4 | Mozambique | Moz2.54 | Mozambique |
| H3.22/32 | Bangladesh | H2.12.11 | Bangladesh | H9.10.4 | Bangladesh | Indo14 | Indonesia | JPN35 | Japan | Moz2.5 | Mozambique | Moz2.55 | Mozambique |
| H3.22/33 | Bangladesh | H5.3/12.1 | Bangladesh | H9.10.5 | Bangladesh | Indo15 | Indonesia | JPN36 | Japan | Moz2.6 | Mozambique | Moz2.56 | Mozambique |
| H3.22/34 | Bangladesh | H5.3/12.2 | Bangladesh | H9.10.6 | Bangladesh | Indo16 | Indonesia | JPN37 | Japan | Moz2.7 | Mozambique | Moz2.57 | Mozambique |
| H3.22/35 | Bangladesh | H5.3/12.3 | Bangladesh | H9.10.7 | Bangladesh | Indo17 | Indonesia | JPN38 | Japan | Moz2.8 | Mozambique | Moz2.58 | Mozambique |
| H3.22/36 | Bangladesh | H5.3/12.4 | Bangladesh | H199024.39/11 | Bangladesh | Indo18 | Indonesia | JPN39 | Japan | Moz2.9 | Mozambique | Moz2.59 | Mozambique |
| H3.22/37 | Bangladesh | H5.3/12.5 | Bangladesh | H5.14/09.30 | Bangladesh | Indo19 | Indonesia | JPN40 | Japan | Moz2.10 | Mozambique | Moz2.60 | Mozambique |
| H3.22/38 | Bangladesh | H5.3/12.6 | Bangladesh | H9.10.9 | Bangladesh | Indo20 | Indonesia | JPN41 | Japan | Moz2.11 | Mozambique | Moz2.61 | Mozambique |
| H3.22/39 | Bangladesh | H5.3/12.7 | Bangladesh | H9.10.10 | Bangladesh | Indo21 | Indonesia | JPN42 | Japan | Moz2.12 | Mozambique | Moz1.1 | Mozambique |
| H3.22/40 | Bangladesh | H5.3/12.8 | Bangladesh | H9.10.11 | Bangladesh | Indo22 | Indonesia | JPN43 | Japan | Moz2.13 | Mozambique | Moz1.2 | Mozambique |
| H6.19/12 | Bangladesh | H11.20/15 | Bangladesh | H9.10.12 | Bangladesh | Indo23 | Indonesia | JPN44 | Japan | Moz2.14 | Mozambique | Moz1.3 | Mozambique |
| H5.14/09.1 | Bangladesh | H5.3/12.9 | Bangladesh | H3.22/6 | Bangladesh | Indo24 | Indonesia | JPN45 | Japan | Moz2.15 | Mozambique | Moz1.4 | Mozambique |
| H5.14/09.2 | Bangladesh | H5.3/12.10 | Bangladesh | H3.22/7 | Bangladesh | Indo25 | Indonesia | JPN46 | Japan | Moz2.16 | Mozambique | Moz1.5 | Mozambique |
| H5.14/09.3 | Bangladesh | H5.3/12.11 | Bangladesh | H3.22/8 | Bangladesh | Indo26 | Indonesia | JPN47 | Japan | Moz2.17 | Mozambique | Moz1.6 | Mozambique |
| H5.14/09.4 | Bangladesh | H5.ej.10 | Bangladesh | H3.22/9 | Bangladesh | Indo27 | Indonesia | JPN48 | Japan | Moz2.18 | Mozambique | Moz1.7 | Mozambique |
| H5.14/09.5 | Bangladesh | H5.3/12.12 | Bangladesh | H3.22/10 | Bangladesh | Indo28 | Indonesia | JPN49 | Japan | Moz2.19 | Mozambique | Moz1.8 | Mozambique |
| H5.14/09.6 | Bangladesh | H5.3/12.13 | Bangladesh | H9.7.12 | Bangladesh | JPN1.1144.2 | Japan | JPN50 | Japan | Moz2.20 | Mozambique | Moz1.10 | Mozambique |
| H5.14/09.7 | Bangladesh | H5.3/12.14 | Bangladesh | H3.22/11 | Bangladesh | JPN9 | Japan | JPN51 | Japan | Moz2.21 | Mozambique | Moz1.11 | Mozambique |
| H5.14/09.8 | Bangladesh | H5.3/12.15 | Bangladesh | H3.22/12 | Bangladesh | JPN2 | Japan | JPN52 | Japan | Moz2.22 | Mozambique | Moz1.12 | Mozambique |
| H5.14/09.9 | Bangladesh | H2.21/11.1 | Bangladesh | H3.22/13 | Bangladesh | JPN1 | Japan | JPN53 | Japan | Moz2.23 | Mozambique | Moz1.13 | Mozambique |
| H5.14/09.10 | Bangladesh | H2.21/11.2 | Bangladesh | H3.22/14 | Bangladesh | JPN3 | Japan | JPN55 | Japan | Moz2.24 | Mozambique | Moz1.14 | Mozambique |
| H6.3/14 | Bangladesh | H2.21/11.3 | Bangladesh | H3.22/15 | Bangladesh | JPN4 | Japan | JPN56 | Japan | Moz2.25 | Mozambique | Moz1.16 | Mozambique |
| H5.14/09.11 | Bangladesh | H9.48.11 | Bangladesh | H3.22/16 | Bangladesh | SPO104 | Japan | JPN5 | Japan | Moz2.26 | Mozambique | Moz1.17 | Mozambique |
| H5.14/09.12 | Bangladesh | H2.21/11.4 | Bangladesh | H3.22/17 | Bangladesh | JPN6 | Japan | Moz1 | Mozambique | Moz2.27 | Mozambique | Moz1.18 | Mozambique |
| H5.14/09.13 | Bangladesh | H2.21/11.5 | Bangladesh | H3.22/18 | Bangladesh | JPN7 | Japan | Moz2 | Mozambique | Moz2.28 | Mozambique | Moz1.19 | Mozambique |
| H5.14/09.14 | Bangladesh | H2.21/11.6 | Bangladesh | H3.22/19 | Bangladesh | JPN8 | Japan | Moz3 | Mozambique | Moz2.29 | Mozambique | Moz1.20 | Mozambique |
| H5.14/09.15 | Bangladesh | H2.21/11.7 | Bangladesh | H3.22/20 | Bangladesh | JPN10 | Japan | Moz4 | Mozambique | Moz2.30 | Mozambique | Moz1.21 | Mozambique |
| H5.14/09.16 | Bangladesh | H2.21/11.8 | Bangladesh | H27.2/12 | Bangladesh | JPN11 | Japan | Moz5 | Mozambique | Moz2.31 | Mozambique | Moz1.22 | Mozambique |
| H5.14/09.17 | Bangladesh | H2.21/11.9 | Bangladesh | H3.22/21 | Bangladesh | JPN12 | Japan | Moz6 | Mozambique | Moz2.32 | Mozambique | Moz1.23 | Mozambique |
| H5.14/09.18 | Bangladesh | H2.21/11.10 | Bangladesh | H3.22/22 | Bangladesh | JPN13 | Japan | Moz7 | Mozambique | Moz2.33 | Mozambique | Moz1.24 | Mozambique |
| H5.14/09.19 | Bangladesh | H2.21/11.11 | Bangladesh | H3.22/23 | Bangladesh | JPN14 | Japan | Moz8 | Mozambique | Moz2.34 | Mozambique | Moz1.25 | Mozambique |
| H5.14/09.20 | Bangladesh | H2.21/11.12 | Bangladesh | H3.22/24 | Bangladesh | JPN15 | Japan | Moz9 | Mozambique | Moz2.35 | Mozambique | Moz1.26 | Mozambique |
| H9.6/11 | Bangladesh | H2.21/11.13 | Bangladesh | H3.22/25 | Bangladesh | JPN16 | Japan | Moz10 | Mozambique | Moz2.36 | Mozambique | Moz1.27 | Mozambique |
| H5.14/09.21 | Bangladesh | H199024.22/11 | Bangladesh | H3.22/26 | Bangladesh | JPN17 | Japan | Moz11 | Mozambique | Moz2.37 | Mozambique | Moz1.28 | Mozambique |
| H5.14/09.22 | Bangladesh | H2.21/11.14 | Bangladesh | H3.22/27 | Bangladesh | JPN18 | Japan | Moz12 | Mozambique | Moz2.38 | Mozambique | Moz1.29 | Mozambique |
| H5.14/09.23 | Bangladesh | H2.21/11.15 | Bangladesh | H3.22/28 | Bangladesh | JPN19 | Japan | Moz13 | Mozambique | Moz2.39 | Mozambique | Moz1.30 | Mozambique |
| H5.14/09.24 | Bangladesh | H2.21/11.16 | Bangladesh | H3.22/29 | Bangladesh | JPN20 | Japan | Moz14 | Mozambique | Moz2.40 | Mozambique | Moz1.31 | Mozambique |
| H5.14/09.25 | Bangladesh | H2.21/11.17 | Bangladesh | H3.22/30 | Bangladesh | JPN21 | Japan | Moz15 | Mozambique | Moz2.41 | Mozambique | Moz1.32 | Mozambique |
| H5.14/09.26 | Bangladesh | H2.21/11.18 | Bangladesh | Indo1 | Indonesia | JPN22 | Japan | Moz16 | Mozambique | Moz2.42 | Mozambique | Moz1.33 | Mozambique |
| H5.14/09.27 | Bangladesh | H2.21/11.19 | Bangladesh | Indo2 | Indonesia | JPN23 | Japan | Moz17 | Mozambique | Moz2.43 | Mozambique | Moz1.34 | Mozambique |
| H5.14/09.28 | Bangladesh | H2.21/11.20 | Bangladesh | Indo3 | Indonesia | JPN24 | Japan | Moz18 | Mozambique | Moz2.44 | Mozambique | Moz1.35 | Mozambique |
| H5.14/09.29 | Bangladesh | H2.21/11.21 | Bangladesh | Indo4 | Indonesia | JPN25 | Japan | Moz19 | Mozambique | Moz2.45 | Mozambique | Moz1.36 | Mozambique |
| H6.52.11 | Bangladesh | H2.21/11.22 | Bangladesh | Indo5 | Indonesia | JPN26 | Japan | Moz20 | Mozambique | Moz2.46 | Mozambique | Moz1.37 | Mozambique |
| H9.9/12 | Bangladesh | H2.21/11.23 | Bangladesh | Indo6 | Indonesia | SPM103 | Japan | Moz21 | Mozambique | Moz2.47 | Mozambique | Moz1.38 | Mozambique |
| Moz1.9 | Mozambique | Moz1.15 | Mozambique | Moz1.41 | Mozambique | Moz1.40 | Mozambique | Moz1.39 | Mozambique |  |  |  |  |

Table S2. Analysis of variance of 351 sweet potato genotypes investigated with four check varieties in an augmented experimental design

| Source of variation | df | Mean sum of square (MSS) | | | | |
| --- | --- | --- | --- | --- | --- | --- |
|  |  | FW | RN | RW | MRN | MRW |
| Genotype (unadjusted) | 354 | 78605^**^ | 13.341^**^ | 110952^**^ | 2.596^*^ | 84155^**^ |
| Block (adjusted) | 9 | 67038** | 4.4152^ns^ | 219264*** | 8.188*** | 204954*** |
| Check | 3 | 161885*** | 13.0252^ns^ | 156124* | 2.294^ns^ | 150795* |
| Genotype (without check) | 350 | 77893*** | 13.3142** | 103796** | 2.409* | 78201** |
| Check + Genotype | 1 | 78150^ns^ | 23.6363^ns^ | 2480134*** | 68.757*** | 1968213*** |
| Residuals | 36 | 19423 | 6.0614 | 50401 | 1.398 | 38331 |
| CV (%) | | 35.5 | 38.9 | 41.8 | 46.3 | 49.2 |

^df^ degrees of freedom, ^CV^ coefficient of variation, ^***^significant at p<0.001, ^**^significant at p<0.01, ^*^significant at p<0.05, ^NS^ non-significant, ^FW^ average foliage fresh weight per plant, ^RN^ average storage root number per plant, ^RW^ average storage root weight per plant, ^MRN^ marketable storage root number per plant and ^MRW^ marketable storage root weight per plant

Table S3. Adjusted mean values for yield and yield contributing traits of 351 sweet potato genotypes, along with four check varieties, in an augmented experimental design at Bogura, Bangladesh during the first season

| Genotypes | FW (g) | RN | RW (g) | MRN | MRW (g) |
| --- | --- | --- | --- | --- | --- |
| BARIMistialu-12^CK^ | 310.52 | 7.29 | 767.83 | 4.25 | 621.92 |
| BARIMistialu-14^CK^ | 327.22 | 6.67 | 621.21 | 3.79 | 495.14 |
| BARIMistialu-15^CK^ | 478.18 | 8.37 | 654.74 | 3.46 | 459.20 |
| BARIMistialu-8^CK^ | 536.75 | 6.02 | 862.61 | 3.31 | 690.53 |
| Deshisada1/19 | 306.84 | 5.91 | 662.03 | 2.92 | 438.51 |
| Deshisada2/12 | 304.27 | 6.64 | 291.57 | 1.53 | 108.97 |
| Deshisada3/14 | 514.91 | 5.68 | 1000.87 | 4.70 | 922.75 |
| H11.15/10 | 174.44 | 7.91 | 540.78 | 2.82 | 352.51 |
| H11.20/15 | 517.84 | 8.16 | 675.53 | 3.42 | 412.76 |
| H16.ej.10 | 516.92 | 10.43 | 811.09 | 4.05 | 463.95 |
| H199024.22/11 | 603.25 | 4.10 | 356.42 | 2.05 | 261.95 |
| H199024.39/11 | 605.84 | 3.91 | 696.78 | 4.42 | 715.51 |
| H2.12.11 | 69.24 | 6.68 | 523.54 | 3.53 | 355.42 |
| H2.21/11 | -94.09 | -0.32 | -36.13 | 0.20 | -17.25 |
| H2.21/11.1 | 681.61 | 7.64 | 524.90 | 2.19 | 220.63 |
| H2.21/11.10 | 354.94 | 8.11 | 737.10 | 3.66 | 513.10 |
| H2.21/11.11 | 115.76 | -0.34 | 241.79 | 0.06 | 189.27 |
| H2.21/11.12 | -17.06 | -0.69 | -156.10 | -0.14 | -126.70 |
| H2.21/11.13 | 861.94 | 10.31 | 647.90 | 4.86 | 379.30 |
| H2.21/11.14 | -17.06 | -0.69 | -156.10 | -0.14 | -126.70 |
| H2.21/11.15 | 957.27 | 9.98 | 818.23 | 2.86 | 453.97 |
| H2.21/11.16 | 457.94 | 4.81 | 329.90 | 1.36 | 146.30 |
| H2.21/11.17 | -17.06 | -0.69 | -156.10 | -0.14 | -126.70 |
| H2.21/11.18 | 383.44 | 7.56 | 324.65 | 1.86 | 128.80 |
| H2.21/11.19 | 358.92 | 2.77 | 476.09 | 3.05 | 493.62 |
| H2.21/11.2 | 176.94 | 2.31 | 77.90 | 0.86 | 49.30 |
| H2.21/11.20 | 600.19 | -0.69 | 372.15 | 2.36 | 197.55 |
| H2.21/11.21 | 244.44 | 5.06 | 302.15 | 3.11 | 264.30 |
| H2.21/11.22 | 333.19 | 4.31 | 232.90 | 1.11 | 45.80 |
| H2.21/11.23 | 348.76 | 5.66 | 469.79 | 1.06 | 281.27 |
| H2.21/11.24 | -94.09 | -0.32 | -36.13 | 0.20 | -17.25 |
| H2.21/11.25 | 305.91 | 3.68 | 338.87 | 2.20 | 254.75 |
| H2.21/11.26 | 155.91 | 5.01 | 295.20 | 1.87 | 189.75 |
| H2.21/11.3 | 324.19 | 6.81 | 367.90 | 2.11 | 188.05 |
| H2.21/11.4 | 752.44 | 7.81 | 457.40 | 0.86 | 274.80 |
| H2.21/11.5 | 1235.94 | 9.31 | 1104.90 | 1.86 | 661.30 |
| H2.21/11.6 | 379.94 | 7.31 | 609.57 | 2.19 | 399.63 |
| H2.21/11.7 | 385.94 | 5.31 | 523.23 | 2.86 | 441.97 |
| H2.21/11.8 | 437.27 | 6.64 | 452.23 | 2.86 | 343.30 |
| H2.21/11.9 | 361.74 | 8.51 | 588.70 | 2.46 | 349.30 |
| H2.34/11 | 666.19 | 7.06 | 874.65 | 2.86 | 662.05 |
| H27.2/12 | 222.84 | 4.51 | 412.58 | 3.22 | 356.31 |
| H3.22/10 | 208.58 | 7.68 | 599.87 | 4.20 | 485.75 |
| H3.22/11 | 516.96 | 4.46 | 580.99 | 2.06 | 435.27 |
| H3.22/12 | 983.41 | 4.18 | 850.37 | 2.20 | 717.75 |
| H3.22/13 | 384.26 | 5.16 | 515.79 | 2.56 | 406.77 |
| H3.22/14 | 447.76 | 3.66 | 726.79 | 4.06 | 674.27 |
| H3.22/15 | 144.66 | 7.68 | 477.37 | 2.20 | 231.00 |
| H3.22/16 | 303.91 | 6.18 | 526.37 | 1.95 | 308.75 |
| H3.22/17 | 279.16 | 3.86 | 444.59 | 2.06 | 368.27 |
| H3.22/18 | 196.24 | 4.68 | 484.20 | 3.87 | 488.08 |
| H3.22/19 | 505.76 | 8.99 | 739.46 | 2.73 | 542.94 |
| H3.22/20 | 232.91 | 5.68 | 416.62 | 2.45 | 287.00 |
| H3.22/21 | 480.31 | 7.28 | 463.07 | 2.00 | 293.55 |
| H3.22/22 | 430.36 | 5.86 | 554.19 | 1.86 | 415.07 |
| H3.22/23 | 258.41 | 7.68 | 265.37 | 1.20 | 139.25 |
| H3.22/24 | 142.41 | 8.18 | 611.37 | 3.20 | 404.00 |
| H3.22/25 | 189.71 | 8.88 | 761.47 | 3.60 | 487.55 |
| H3.22/26 | 131.91 | 4.68 | 763.87 | 5.20 | 782.75 |
| H3.22/27 | 386.11 | 7.08 | 420.27 | 1.60 | 155.15 |
| H3.22/28 | 347.01 | 9.66 | 615.04 | 2.06 | 406.52 |
| H3.22/29 | 239.41 | 9.93 | 720.62 | 2.95 | 411.75 |
| H3.22/30 | 261.76 | 3.16 | 304.29 | 0.06 | 189.27 |
| H3.22/31 | 133.91 | 6.68 | 59.87 | 0.20 | -17.25 |
| H3.22/32 | 500.43 | 4.99 | 409.79 | 0.73 | 254.60 |
| H3.22/33 | 179.58 | 11.35 | 1025.20 | 4.20 | 691.75 |
| H3.22/34 | 631.76 | 6.66 | 744.79 | 3.56 | 614.77 |
| H3.22/35 | 296.76 | 4.66 | 509.29 | 1.06 | 311.27 |
| H3.22/36 | 140.58 | 8.35 | 755.54 | 2.87 | 389.08 |
| H3.22/37 | 314.01 | 3.41 | 357.29 | 0.81 | 236.02 |
| H3.22/38 | 31.91 | 1.68 | -4.13 | 0.20 | -17.25 |
| H3.22/39 | 493.43 | 5.33 | 450.46 | 1.39 | 296.60 |
| H3.22/40 | 41.78 | 0.60 | -20.16 | 0.33 | -35.24 |
| H3.22/6 | 263.91 | 8.01 | 503.87 | 2.87 | 285.08 |
| H3.22/7 | 431.76 | 5.16 | 547.79 | 2.56 | 406.77 |
| H3.22/8 | 10.91 | 4.68 | 374.87 | 3.20 | 344.75 |
| H3.22/9 | 242.91 | 5.68 | 708.87 | 5.20 | 705.75 |
| H4.2/12 | 1037.94 | 7.31 | 656.90 | 2.61 | 459.05 |
| H5.14/09.1 | 152.78 | 8.10 | 315.84 | 1.83 | 139.76 |
| H5.14/09.10 | 413.03 | 7.85 | 558.59 | 3.83 | 389.26 |
| H5.14/09.11 | 304.76 | 6.16 | 751.79 | 3.56 | 607.77 |
| H5.14/09.12 | 369.76 | 5.66 | 839.79 | 5.06 | 766.27 |
| H5.14/09.13 | 191.11 | 6.68 | 443.47 | 3.00 | 309.55 |
| H5.14/09.14 | 281.78 | 5.27 | 534.17 | 3.33 | 437.43 |
| H5.14/09.15 | -73.22 | 0.60 | -20.16 | 0.33 | -35.24 |
| H5.14/09.16 | 186.76 | 0.37 | 35.88 | -0.53 | -17.40 |
| H5.14/09.17 | 174.53 | 6.60 | 750.59 | 3.33 | 534.01 |
| H5.14/09.18 | 26.78 | 2.60 | 255.84 | 1.33 | 212.76 |
| H5.14/09.19 | 265.76 | 2.87 | 214.88 | 1.47 | 158.60 |
| H5.14/09.2 | 230.45 | 7.60 | 759.84 | 5.00 | 662.76 |
| H5.14/09.20 | 611.26 | 8.87 | 587.38 | 2.47 | 418.60 |
| H5.14/09.21 | 387.38 | 7.40 | 1031.84 | 4.53 | 798.56 |
| H5.14/09.22 | 274.76 | 4.37 | 79.88 | -0.53 | -17.40 |
| H5.14/09.23 | 703.09 | 0.37 | 35.88 | -0.53 | -17.40 |
| H5.14/09.24 | 484.09 | 8.04 | 223.21 | 0.80 | 60.60 |
| H5.14/09.25 | 690.09 | 3.04 | 359.21 | 0.47 | 258.60 |
| H5.14/09.26 | 543.76 | 6.70 | 815.88 | 4.14 | 675.60 |
| H5.14/09.27 | 265.76 | 6.37 | 479.88 | 1.47 | 317.60 |
| H5.14/09.28 | 548.76 | 2.87 | 38.88 | 0.47 | 141.60 |
| H5.14/09.29 | 715.26 | 9.37 | 872.88 | 3.97 | 672.60 |
| H5.14/09.3 | 746.76 | 0.66 | 256.79 | 0.06 | 189.27 |
| H5.14/09.30 | 115.76 | -0.34 | 241.79 | 0.06 | 189.27 |
| H5.14/09.4 | 479.26 | 6.16 | 673.79 | 3.06 | 547.27 |
| H5.14/09.5 | 400.01 | 12.91 | 773.54 | 2.81 | 480.02 |
| H5.14/09.6 | 507.78 | 13.60 | 459.84 | 1.33 | 156.76 |
| H5.14/09.7 | 692.76 | 8.66 | 1040.79 | 3.56 | 801.27 |
| H5.14/09.8 | 474.76 | 10.33 | 762.12 | 3.06 | 486.60 |
| H5.14/09.9 | 360.78 | 8.10 | 639.84 | 4.08 | 490.51 |
| H5.3/12.1 | -17.06 | -0.69 | -156.10 | -0.14 | -126.70 |
| H5.3/12.10 | 511.24 | 5.35 | 564.54 | 2.87 | 451.75 |
| H5.3/12.11 | 489.26 | 4.66 | 639.79 | 2.06 | 502.77 |
| H5.3/12.12 | 129.24 | 4.35 | 288.87 | 1.53 | 192.08 |
| H5.3/12.13 | 589.76 | 5.16 | 622.29 | 3.06 | 518.27 |
| H5.3/12.14 | 25.91 | 5.68 | 468.87 | 3.20 | 300.75 |
| H5.3/12.15 | 463.94 | 6.56 | 473.15 | 2.61 | 316.55 |
| H5.3/12.2 | 605.61 | 6.31 | 663.23 | 2.86 | 469.30 |
| H5.3/12.3 | 466.44 | 7.56 | 450.90 | 1.36 | 192.55 |
| H5.3/12.4 | 383.00 | 6.85 | 585.34 | 2.88 | 449.12 |
| H5.3/12.5 | 394.19 | 7.06 | 435.40 | 1.61 | 143.80 |
| H5.3/12.6 | 412.76 | 2.66 | 449.79 | 2.06 | 328.27 |
| H5.3/12.7 | 395.76 | 2.66 | 549.79 | 3.06 | 497.27 |
| H5.3/12.8 | 115.76 | -0.34 | 241.79 | 0.06 | 189.27 |
| H5.3/12.9 | 297.84 | 2.91 | 137.78 | 1.42 | 127.51 |
| H5.ej.10 | 927.07 | 11.48 | 1017.77 | 3.12 | 789.03 |
| H6.19/12 | 477.45 | 6.90 | 820.69 | 4.98 | 744.42 |
| H6.3/14 | 600.92 | 5.10 | 736.76 | 4.05 | 671.29 |
| H6.52.11 | 738.26 | 4.87 | 909.88 | 3.47 | 836.60 |
| H9.10.1 | 488.44 | 10.31 | 516.40 | 2.36 | 171.80 |
| H9.10.10 | 231.91 | 5.68 | 278.87 | 1.20 | 153.75 |
| H9.10.11 | 639.24 | 5.68 | 488.87 | 2.53 | 356.08 |
| H9.10.12 | 1199.91 | 7.18 | 1336.87 | 5.20 | 1266.25 |
| H9.10.2 | 445.26 | 3.66 | 417.79 | 1.56 | 291.27 |
| H9.10.3 | 385.44 | 6.56 | 172.40 | 0.86 | 14.30 |
| H9.10.4 | 228.91 | 4.18 | 465.37 | 3.20 | 456.75 |
| H9.10.5 | 625.94 | 7.64 | 653.90 | 3.19 | 445.97 |
| H9.10.6 | 337.56 | 4.46 | 457.79 | 1.86 | 328.67 |
| H9.10.7 | 105.91 | 2.68 | 10.87 | 0.20 | -17.25 |
| H9.10.9 | 493.94 | 8.31 | 551.23 | 3.19 | 302.97 |
| H9.13 | 231.17 | 7.24 | 423.78 | 3.09 | 288.84 |
| H9.32/11 | 139.91 | 4.01 | 272.87 | 2.20 | 247.42 |
| H9.48.11 | 871.58 | 10.43 | 1042.09 | 4.71 | 769.62 |
| H9.6/11 | 521.79 | 8.06 | 649.90 | 2.86 | 455.05 |
| H9.7.12 | 1125.61 | 7.98 | 587.23 | 2.53 | 363.97 |
| H9.9/12 | 222.76 | 5.66 | 527.29 | 2.06 | 392.27 |
| Indo1 | 592.34 | 8.66 | 878.53 | 5.17 | 749.76 |
| Indo10 | 685.84 | 7.58 | 753.45 | 3.42 | 575.18 |
| Indo11 | 344.25 | 6.85 | 947.09 | 4.13 | 810.37 |
| Indo12 | 348.17 | 4.24 | 430.45 | 2.09 | 359.84 |
| Indo13 | 408.59 | 6.41 | 336.53 | 1.67 | 214.01 |
| Indo14 | 395.59 | 3.91 | 691.78 | 3.42 | 664.76 |
| Indo15 | 767.84 | 7.91 | 514.78 | 2.42 | 252.51 |
| Indo16 | 276.25 | 5.10 | 401.09 | 3.05 | 374.29 |
| Indo17 | 549.58 | 6.43 | 454.42 | 2.71 | 313.62 |
| Indo18 | 254.84 | 2.91 | 156.78 | 1.42 | 115.51 |
| Indo19 | 515.51 | 7.58 | 1018.11 | 5.09 | 921.84 |
| Indo2 | 209.84 | 7.16 | 525.53 | 2.67 | 329.26 |
| Indo20 | 904.34 | 7.91 | 735.78 | 2.42 | 541.01 |
| Indo21 | 12.84 | -0.09 | 3.78 | 0.42 | 22.51 |
| Indo22 | 620.34 | 6.41 | 599.78 | 1.42 | 316.51 |
| Indo23 | 609.51 | 3.91 | 739.78 | 3.42 | 699.84 |
| Indo24 | 519.34 | 5.41 | 460.53 | 2.17 | 337.51 |
| Indo25 | 356.45 | 5.50 | 558.09 | 3.58 | 465.82 |
| Indo26 | 626.84 | 1.91 | 742.78 | 2.42 | 761.51 |
| Indo27 | 735.09 | 7.41 | 570.53 | 2.67 | 379.01 |
| Indo28 | 277.00 | 4.10 | 409.34 | 1.88 | 281.62 |
| Indo3 | 308.34 | 5.41 | 773.53 | 4.17 | 687.76 |
| Indo4 | -208.93 | -2.52 | -418.23 | -1.88 | -358.97 |
| Indo5 | 51.07 | 3.48 | 108.27 | 1.12 | 87.03 |
| Indo6 | 373.84 | 3.91 | 279.78 | 1.42 | 163.51 |
| Indo7 | 349.84 | 4.91 | 516.28 | 3.42 | 438.01 |
| Indo8 | 544.84 | 4.41 | 161.78 | 0.42 | 22.51 |
| Indo9 | 413.17 | 3.91 | 566.45 | 3.42 | 548.18 |
| JPN1 | 606.83 | 2.49 | 665.79 | 2.29 | -8.20 |
| JPN1.1144.2 | 532.28 | 9.10 | 954.84 | 3.33 | 733.26 |
| JPN10 | 537.03 | 13.29 | 708.59 | 3.49 | 412.80 |
| JPN11 | 284.53 | 5.29 | 716.09 | 1.99 | 530.80 |
| JPN12 | 294.78 | 10.29 | 764.59 | 2.99 | 510.80 |
| JPN13 | 917.03 | 5.29 | 449.59 | 1.49 | 351.80 |
| JPN14 | 391.92 | 9.43 | 888.42 | 4.05 | 629.29 |
| JPN15 | 979.03 | 3.62 | 810.59 | 2.49 | 751.47 |
| JPN16 | 627.40 | 2.15 | 448.10 | 1.79 | 445.36 |
| JPN17 | 636.07 | -0.52 | 90.10 | 0.12 | 149.36 |
| JPN18 | 240.57 | 1.98 | 65.77 | 0.12 | 53.53 |
| JPN19 | -208.93 | -2.52 | -418.23 | -1.88 | -358.97 |
| JPN2 | 121.00 | 9.35 | 849.09 | 4.63 | 689.62 |
| JPN20 | 339.07 | 10.81 | 483.44 | 2.12 | 184.03 |
| JPN21 | -208.93 | -2.52 | -418.23 | -1.88 | -358.97 |
| JPN22 | 269.32 | 8.48 | 289.77 | 1.62 | 138.78 |
| JPN23 | 1091.07 | 6.48 | 273.77 | 1.12 | 206.03 |
| JPN24 | 107.07 | 8.98 | -70.23 | -0.38 | -175.97 |
| JPN25 | 154.32 | 6.73 | 313.02 | 2.12 | 200.53 |
| JPN26 | 3.07 | 2.48 | 13.77 | 0.12 | -29.97 |
| JPN28 | 112.32 | 7.48 | 461.52 | 2.12 | 190.53 |
| JPN29 | 62.07 | 2.81 | -270.56 | -1.21 | -275.30 |
| JPN3 | 598.70 | 1.96 | 699.59 | 1.82 | 680.80 |
| JPN30 | 251.57 | 8.98 | 238.77 | 1.62 | 72.53 |
| JPN31 | -17.26 | 2.81 | -239.23 | -1.21 | -285.97 |
| JPN32 | 281.07 | 2.48 | 196.77 | 3.12 | 256.03 |
| JPN33 | 106.07 | 2.48 | -2.23 | 1.45 | -14.30 |
| JPN34 | -208.93 | -2.52 | -418.23 | -1.88 | -358.97 |
| JPN35 | 139.57 | 9.48 | 386.77 | 2.62 | 177.03 |
| JPN36 | 184.57 | 7.98 | 506.27 | 3.12 | 222.03 |
| JPN37 | 457.07 | 8.48 | 790.77 | 5.12 | 663.03 |
| JPN38 | 611.40 | 6.48 | 203.10 | 0.12 | -94.97 |
| JPN39 | 328.25 | 6.77 | 997.42 | 4.71 | 878.62 |
| JPN4 | -20.97 | -0.71 | 4.59 | -0.51 | -8.20 |
| JPN40 | 92.57 | 4.98 | 135.27 | 3.12 | 104.03 |
| JPN41 | 129.07 | 7.23 | 417.52 | 3.37 | 361.78 |
| JPN42 | 226.82 | 11.48 | 226.77 | 0.87 | -65.47 |
| JPN43 | 101.07 | 0.48 | 14.27 | -0.38 | -2.97 |
| JPN44 | 55.47 | 4.68 | 196.97 | 1.32 | 98.63 |
| JPN45 | 144.40 | 9.15 | -53.56 | -1.88 | -358.97 |
| JPN46 | -208.93 | -2.52 | -418.23 | -1.88 | -358.97 |
| JPN47 | 3.07 | 4.73 | 115.27 | 0.87 | -7.72 |
| JPN48 | 357.84 | 1.91 | 378.78 | 1.42 | 278.51 |
| JPN49 | 356.59 | 7.66 | 452.28 | 3.17 | 282.51 |
| JPN5 | 519.84 | 6.91 | 552.11 | 3.09 | 403.18 |
| JPN50 | 574.09 | 10.41 | 784.78 | 4.42 | 511.51 |
| JPN51 | 314.84 | 1.91 | 75.78 | 0.42 | 22.51 |
| JPN52 | 370.84 | 8.16 | 551.78 | 2.67 | 337.01 |
| JPN53 | 496.84 | 7.41 | 759.28 | 4.42 | 597.51 |
| JPN55 | 1075.34 | 6.91 | 453.28 | 3.42 | 386.51 |
| JPN56 | 26.07 | 1.48 | 90.77 | 0.79 | 105.03 |
| JPN6 | 308.03 | 5.79 | 1093.59 | 5.49 | 1063.30 |
| JPN7 | 1105.70 | 8.96 | 882.26 | 4.16 | 690.80 |
| JPN8 | 898.78 | 5.54 | 845.84 | 2.74 | 747.55 |
| JPN9 | 416.94 | 9.81 | 454.65 | 1.36 | 87.05 |
| Moz1 | 326.28 | 6.10 | 603.34 | 2.83 | 484.76 |
| Moz1.1 | -25.22 | -0.32 | -251.25 | -2.84 | -297.86 |
| Moz1.10 | -20.97 | -0.71 | 4.59 | -0.51 | -8.20 |
| Moz1.11 | 389.78 | 11.29 | 831.59 | 4.49 | 595.30 |
| Moz1.12 | 227.65 | 7.90 | 487.89 | 2.58 | 337.02 |
| Moz1.13 | 108.28 | 8.29 | 651.09 | 4.49 | 537.30 |
| Moz1.14 | 278.58 | 18.43 | 847.09 | 3.38 | 406.62 |
| Moz1.15 | 1361.03 | 5.79 | 1676.59 | 4.99 | 1642.30 |
| Moz1.16 | 124.03 | 5.96 | 228.92 | 0.16 | 51.47 |
| Moz1.17 | 221.45 | 7.10 | 569.89 | 3.38 | 448.22 |
| Moz1.18 | 668.03 | 4.29 | 763.59 | 1.49 | 601.80 |
| Moz1.19 | 410.92 | 13.10 | 948.09 | 5.71 | 677.95 |
| Moz1.2 | 186.76 | 0.37 | 35.88 | -0.53 | -17.40 |
| Moz1.20 | 35.53 | 4.79 | 77.59 | -0.51 | -8.20 |
| Moz1.21 | -20.97 | -0.71 | 4.59 | -0.51 | -8.20 |
| Moz1.22 | 287.70 | 7.96 | 481.59 | 1.82 | 221.13 |
| Moz1.23 | 485.78 | 13.43 | 621.50 | 3.66 | 260.14 |
| Moz1.24 | 337.50 | 10.60 | 787.09 | 4.38 | 551.62 |
| Moz1.25 | 820.53 | 3.29 | 509.59 | 1.99 | 431.80 |
| Moz1.26 | 339.75 | 11.10 | 1347.59 | 5.88 | 1018.12 |
| Moz1.27 | 290.03 | 14.29 | 1002.59 | 5.49 | 577.80 |
| Moz1.28 | -25.22 | -0.32 | -251.25 | -2.84 | -297.86 |
| Moz1.29 | 488.25 | 7.43 | 1037.09 | 4.38 | 859.29 |
| Moz1.3 | -25.22 | -0.32 | -251.25 | -2.84 | -297.86 |
| Moz1.30 | -20.97 | -0.71 | 4.59 | -0.51 | -8.20 |
| Moz1.31 | 426.36 | 6.62 | 780.59 | 4.16 | 649.13 |
| Moz1.32 | 275.53 | 7.79 | 789.59 | 5.49 | 1196.80 |
| Moz1.33 | -20.97 | -0.71 | 4.59 | -0.51 | -8.20 |
| Moz1.34 | 377.03 | 4.29 | 364.09 | 0.99 | 193.80 |
| Moz1.35 | 509.36 | 5.62 | 616.59 | 2.49 | 498.47 |
| Moz1.36 | 601.36 | 6.62 | 555.92 | 1.49 | 263.80 |
| Moz1.37 | 226.36 | 5.29 | 484.26 | 2.49 | 395.80 |
| Moz1.38 | 178.36 | 5.96 | 514.59 | 2.16 | 378.80 |
| Moz1.39 | 165.53 | 4.79 | 499.59 | 2.99 | 286.80 |
| Moz1.4 | 424.78 | 4.35 | 438.75 | 1.49 | 362.81 |
| Moz1.40 | 483.03 | 1.29 | 433.59 | 1.49 | 420.80 |
| Moz1.41 | -20.97 | -0.71 | 4.59 | -0.51 | -8.20 |
| Moz1.5 | 114.78 | 4.68 | 329.75 | 1.16 | 250.14 |
| Moz1.6 | -25.22 | -0.32 | -251.25 | -2.84 | -297.86 |
| Moz1.7 | 490.78 | 9.68 | 1087.75 | 2.16 | 942.14 |
| Moz1.8 | 439.82 | 13.73 | 692.02 | 2.12 | 216.78 |
| Moz1.9 | 1067.36 | 2.62 | 656.59 | 1.49 | 597.13 |
| Moz10 | 597.76 | 11.37 | 535.38 | 0.97 | 205.10 |
| Moz11 | 309.76 | 2.66 | 397.79 | 1.06 | 263.27 |
| Moz12 | 477.26 | 13.37 | 699.13 | 3.22 | 340.85 |
| Moz13 | 590.26 | 7.87 | 486.88 | 2.47 | 331.60 |
| Moz14 | 439.03 | 14.35 | 1364.34 | 4.33 | 877.01 |
| Moz15 | 188.45 | 7.93 | 399.51 | 2.00 | 146.76 |
| Moz16 | 284.45 | 9.60 | 1120.84 | 4.33 | 858.43 |
| Moz17 | 139.28 | 7.10 | 500.34 | 2.33 | 265.76 |
| Moz18 | 596.16 | 10.17 | 977.88 | 3.47 | 771.20 |
| Moz19 | 186.76 | 0.37 | 35.88 | -0.53 | -17.40 |
| Moz2 | 536.76 | 11.87 | 918.88 | 3.97 | 624.10 |
| Moz2.10 | 348.76 | 5.70 | 471.21 | 2.47 | 386.93 |
| Moz2.11 | 411.78 | 10.68 | 1144.75 | 4.16 | 917.14 |
| Moz2.12 | 784.78 | 14.68 | 188.75 | 1.16 | -2.86 |
| Moz2.13 | 711.78 | 7.68 | 440.75 | 1.16 | 314.14 |
| Moz2.14 | 304.45 | 19.35 | 59.08 | 0.16 | -29.53 |
| Moz2.15 | 529.78 | 3.01 | 192.75 | -0.84 | 117.47 |
| Moz2.16 | 356.78 | 7.60 | 1068.09 | 4.58 | 906.01 |
| Moz2.17 | 321.28 | 6.60 | 275.34 | 1.33 | 148.76 |
| Moz2.18 | 412.78 | 3.93 | 366.17 | 2.00 | 284.43 |
| Moz2.19 | 202.78 | 10.68 | 490.75 | 3.16 | 319.14 |
| Moz2.2 | 998.76 | 4.37 | 956.88 | 1.47 | 830.60 |
| Moz2.20 | 423.11 | 6.60 | 396.17 | 3.00 | 267.76 |
| Moz2.21 | 435.78 | 5.60 | 316.84 | 2.33 | 199.76 |
| Moz2.22 | 519.78 | 5.27 | 647.84 | 3.33 | 571.09 |
| Moz2.23 | 446.28 | 6.60 | 474.34 | 2.33 | 339.26 |
| Moz2.24 | 816.78 | 3.68 | 568.75 | 0.16 | 510.14 |
| Moz2.25 | 774.78 | 7.68 | 509.75 | 1.16 | 347.64 |
| Moz2.26 | -73.22 | 0.60 | -20.16 | 0.33 | -35.24 |
| Moz2.27 | 1694.78 | 3.68 | 986.75 | 1.16 | 940.14 |
| Moz2.28 | 610.78 | 6.68 | 196.75 | -0.84 | -58.86 |
| Moz2.29 | 43.78 | 5.68 | -134.25 | -2.84 | -297.86 |
| Moz2.3 | 446.03 | 10.85 | 991.59 | 5.08 | 748.01 |
| Moz2.30 | 315.78 | 15.60 | 1200.84 | 5.33 | 746.76 |
| Moz2.31 | 338.03 | 11.10 | 728.59 | 3.33 | 387.26 |
| Moz2.32 | 479.11 | 9.35 | 454.08 | 0.16 | 151.81 |
| Moz2.33 | 431.28 | 9.60 | 694.34 | 3.83 | 472.26 |
| Moz2.34 | 314.43 | 3.87 | 438.38 | 1.47 | 342.60 |
| Moz2.35 | 390.45 | 4.60 | 357.17 | 1.33 | 258.76 |
| Moz2.36 | 268.28 | 7.68 | 214.75 | 0.16 | 38.14 |
| Moz2.37 | 308.28 | 11.60 | 685.84 | 2.83 | 285.26 |
| Moz2.38 | 202.78 | 6.68 | -61.25 | -0.84 | -154.86 |
| Moz2.39 | 269.28 | 13.10 | 815.34 | 4.58 | 426.26 |
| Moz2.4 | 428.76 | 7.04 | 778.55 | 3.47 | 652.27 |
| Moz2.40 | 273.11 | 6.35 | 162.42 | -0.51 | 29.14 |
| Moz2.41 | 99.78 | 1.68 | -119.75 | -1.84 | -161.36 |
| Moz2.42 | 215.78 | 7.43 | -93.25 | -2.34 | -255.86 |
| Moz2.43 | 452.78 | 3.93 | 237.17 | 1.33 | 133.43 |
| Moz2.44 | 433.53 | 14.68 | 156.75 | -0.84 | -120.11 |
| Moz2.45 | 522.53 | 9.35 | 430.59 | 1.33 | 92.26 |
| Moz2.46 | 832.78 | 3.68 | 163.75 | -0.84 | 102.14 |
| Moz2.47 | 125.25 | 5.10 | 270.09 | 1.38 | 176.62 |
| Moz2.48 | 1072.78 | 5.68 | 217.75 | 1.16 | 94.14 |
| Moz2.49 | 232.28 | 10.68 | 358.75 | 1.66 | 127.14 |
| Moz2.5 | 115.76 | -0.34 | 241.79 | 0.06 | 189.27 |
| Moz2.50 | 529.45 | 8.35 | 150.75 | -0.84 | -94.86 |
| Moz2.51 | 501.00 | 8.10 | 457.59 | 2.63 | 305.12 |
| Moz2.52 | 269.58 | 8.48 | -9.85 | -2.04 | -212.66 |
| Moz2.53 | 348.27 | 8.64 | 255.57 | 1.86 | 105.97 |
| Moz2.54 | 238.58 | 6.28 | 212.35 | 1.36 | 100.74 |
| Moz2.55 | 396.44 | 8.81 | 436.40 | 3.86 | 267.30 |
| Moz2.56 | 154.25 | 7.10 | 303.09 | 1.38 | 176.62 |
| Moz2.57 | 255.00 | 5.35 | 475.59 | 3.13 | 425.62 |
| Moz2.58 | 1294.78 | 6.68 | 828.75 | 0.16 | 552.14 |
| Moz2.59 | -25.22 | -0.32 | -251.25 | -2.84 | -297.86 |
| Moz2.6 | 510.26 | 11.87 | 701.88 | 3.47 | 437.10 |
| Moz2.60 | -25.22 | -0.32 | -251.25 | -2.84 | -297.86 |
| Moz2.61 | -25.22 | -0.32 | -251.25 | -2.84 | -297.86 |
| Moz2.7 | 348.76 | 11.37 | 504.88 | 3.47 | 293.60 |
| Moz2.8 | 266.76 | 4.37 | 271.88 | 2.47 | 209.60 |
| Moz2.9 | 186.76 | 0.37 | 35.88 | -0.53 | -17.40 |
| Moz20 | 401.43 | 3.66 | 460.79 | 2.06 | 349.27 |
| Moz21 | 385.76 | 2.66 | 486.79 | 2.06 | 406.77 |
| Moz22 | 186.76 | 0.37 | 35.88 | -0.53 | -17.40 |
| Moz23 | 214.45 | 9.60 | 756.17 | 2.66 | 395.76 |
| Moz24 | 334.78 | 7.60 | 1013.84 | 4.66 | 817.43 |
| Moz25 | 1206.76 | 5.37 | 827.88 | 2.47 | 725.60 |
| Moz3 | 408.51 | 8.12 | 611.38 | 2.22 | 416.60 |
| Moz4 | 328.76 | 5.04 | 484.21 | 2.14 | 354.60 |
| Moz5 | 329.43 | 5.04 | 399.88 | 1.47 | 274.60 |
| Moz6 | 406.76 | 11.04 | 589.55 | 2.80 | 296.93 |
| Moz7 | 372.01 | 6.12 | 609.38 | 2.97 | 393.35 |
| Moz8 | 186.76 | 0.37 | 35.88 | -0.53 | -17.40 |
| Moz9 | 186.76 | 0.37 | 35.88 | -0.53 | -17.40 |
| SPM103 | 125.32 | 8.98 | 462.52 | 4.12 | 323.53 |
| SPO104 | 1081.03 | 2.79 | 660.09 | 2.99 | 897.30 |
| Significance | ** | ** | ** | * | ** |

^CK^ check variety, **significant at p<0.01, *significant at p<0.05, ^FW^ average foliage fresh weight per plant, ^RN^ average storage root number per plant, ^RW^ average storage root weight per plant, ^MRN^ marketable storage root number per plant and ^MRW^ marketable storage root weight per plant

Table S4. Analysis of variance of studied agronomic traits for four source countries

| Source of variations | Degrees of freedom | Mean sum of squares | | | | |
| --- | --- | --- | --- | --- | --- | --- |
|  |  | FW | RN | RW | MRN | MRW |
| Country (adjusted) | 3 | 327852.1 | 66.71 | 744811.3 | 31.95* | 857410.08* |
| Country (unadjusted) | 3 | 397671.3 | 75.12 | 337819.3 | 12.69* | 147907.06* |
| Replication (unadjusted) | 9 | 2050899* | 203.72 | 3483375* | 40.57* | 1828176.25* |
| Replication (adjusted) | 9 | 1981080* | 195.32 | 3890367* | 59.82* | 2537679.27* |
| Error | 387 | 26750162 | 4710.21 | 38836603 | 970.43 | 30329908.92 |
| Total | 399 | 29128913 | 4980.64 | 43064789 | 1042.95 | 33015495.25 |

*Significant at p<0.05, ^FW^ average foliage fresh weight per plant, ^RN^ average storage root number per plant, ^RW^ average storage root weight per plant, ^MRN^ marketable storage root number per plant and ^MRW^ marketable storage root weight per plant

Table S5. Mean values of source countries for studied agronomic traits in Bogura, Bangladesh

| Source country | FW | RN | RW | MRN | MRW |
| --- | --- | --- | --- | --- | --- |
| Bangladesh | 1238.49 | 19.97 | 1697.34 | 8.06 | 1257 |
| Indonesia | 1161.02 | 18.72 | 1586.6 | 7.58 | 1179.86 |
| Japan | 1156.78 | 18.77 | 1584.52 | 7.6 | 1177.29 |
| Mozambique | 1225.37 | 19.73 | 1678.89 | 7.96 | 1243.1 |

^FW^ average foliage fresh weight per plant, ^RN^ average storage root number per plant, ^RW^ average storage root weight per plant, ^MRN^ marketable storage root number per plant and ^MRW^ marketable storage root weight per plant
